# Parity and heart failure risk in diabetic women with coronary artery disease: a multicentre cohort study

**DOI:** 10.1093/eschf/xvag206

**Published:** 2026-08-19

**Authors:** Yuko Fujita, Takeshi Morimoto, Koichi Node, Keiko Mekaru, Shinichiro Ueda

**Affiliations:** Center for Clinical Research Support, Education and Management, University Hospital of the Ryukyus, Okinawa, Japan; Department of Data Science, Hyogo Medical University, Hyogo, Japan; Department of Cardiovascular Medicine, Saga University, Saga, Japan; Perinatal Medical Center and Department of Obstetrics and Gynecology, University Hospital of the Ryukyus, Ginowan, Japan; Heart Failure Initiative, MEXT Program for the Promotion of Clinical Research, University Hospital of the Ryukyus, 1076 Kiyuna, Ginowan, Okinawa 901-2725, Japan

**Keywords:** Parity, Heart failure, Cardiovascular disease, Type 2 diabetes mellitus, Coronary artery disease

## Abstract

**Introduction:**

Women with type 2 diabetes mellitus (T2DM) and coronary artery disease (CAD) face elevated heart failure (HF) risk compared with men. Although population-based studies have linked parity to cardiovascular outcomes, evidence is limited among women with established cardiovascular disease. We investigated the association between parity and HF hospitalization and mortality in women with T2DM and CAD.

**Methods:**

This multicentre retrospective cohort study enrolled 2314 women with T2DM and CAD from 70 hospitals between 2005 and 2022 in Japan. Participants were stratified by parity and followed for a median of 2297 days. Outcomes included HF hospitalization, all-cause mortality, acute myocardial infarction, and stroke. Cox proportional hazards models adjusted for age, cardiovascular risk factors, and medication use.

**Results:**

Women with ≥5 births had significantly higher incidences of HF hospitalization (4.90 vs 3.03 per 100 person-years) and mortality (4.85 vs 3.76 per 100 person-years) compared with <5 births. After adjustment, high parity (≥5 births) was significantly associated with HF hospitalization [hazard ratio (HR): 1.41, 95% confidence interval (CI): 1.04–1.90] but not with mortality (HR: 1.22, 95% CI: .92–1.62). No associations were observed with acute myocardial infarction or stroke.

**Conclusion:**

Among women with T2DM and CAD, high parity independently and outcome specifically predicted HF hospitalization, regardless of accumulated cardiovascular risk factors. Reproductive history should be considered in HF risk stratification even for high-risk women.

## Graphical Abstract

**Graphical Abstract:**
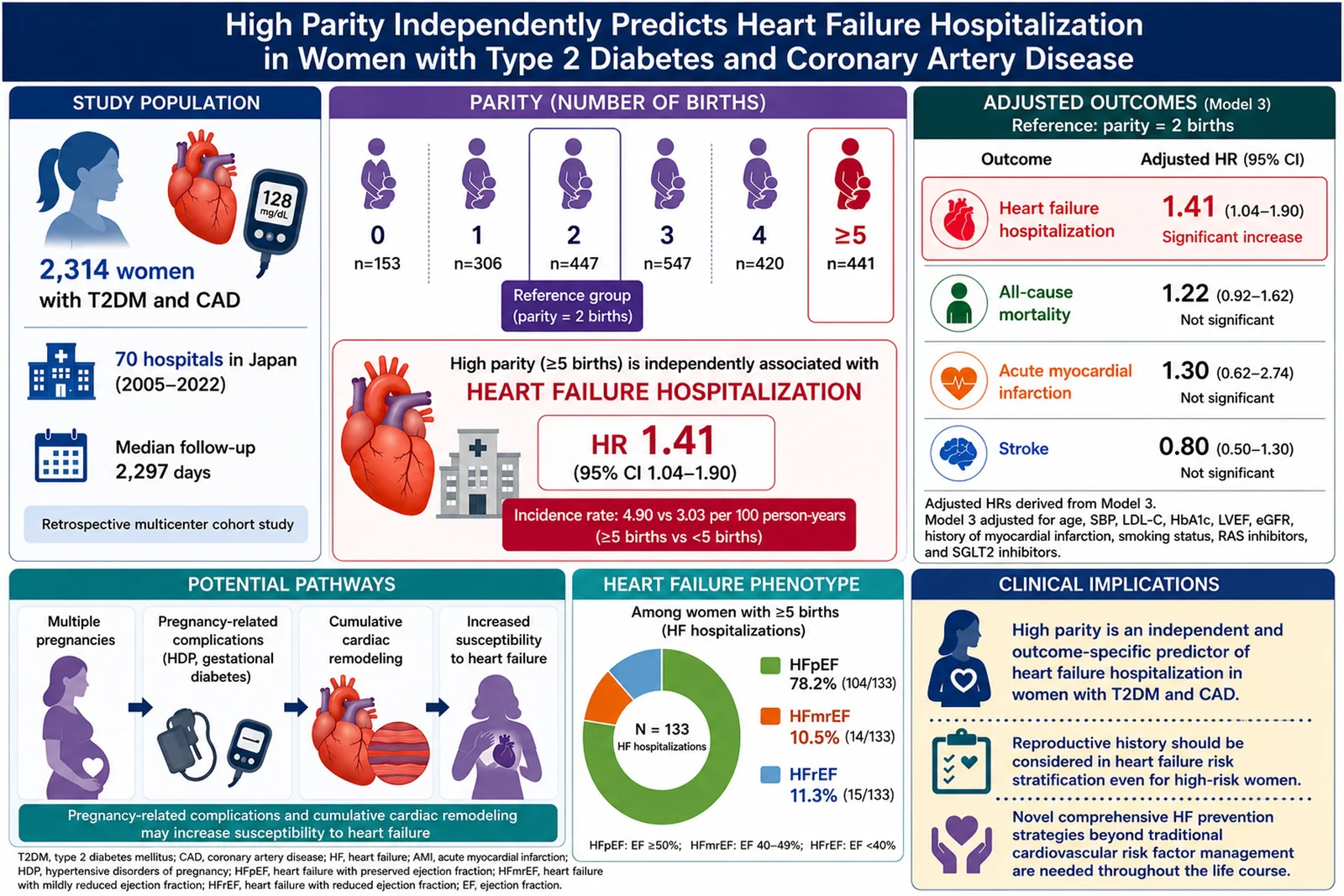
Graphical abstract showing that, among 2314 women with type 2 diabetes and coronary artery disease, high parity (≥5 births) was independently associated with heart failure hospitalization (adjusted HR 1.41, 95% CI 1.04-1.90) but not with other cardiovascular outcomes, suggesting reproductive history should inform heart failure risk stratification.

## Introduction

The global prevalence of diabetes mellitus is increasing at an alarming rate, with projections indicating that more than 783 million individuals will be living with type 2 diabetes mellitus (T2DM) by 2045.^1^ This escalating global trend presents a significant public health challenge, largely due to the associated increase in heart failure (HF) burden. Diabetes mellitus adversely affects cardiac structure and function, partly through the development of diabetic cardiomyopathy, and increases the risk of cardiovascular disease (CVD) and HF by two- to five-fold.^2–6^

The growing number of individuals with diabetes raises serious concern about an impending HF pandemic in Japan, a rapidly ageing society. Among patients with both T2DM and coronary artery disease (CAD), HF occurs more frequently than other major cardiovascular events, including acute myocardial infarction (AMI) and stroke.^5,7^ Furthermore, our previous study demonstrated that women with both T2DM and CAD have a significantly higher risk of developing HF compared with men.^8^ This finding is consistent with sex-specific differences in HF risk observed in prior epidemiological studies, including the Framingham Heart Study.^9,10^ The mechanisms underlying this sex disparity remain incompletely understood. However, several studies suggest that female-specific factors, including pregnancy, childbirth, and menopause, may contribute to long-term cardiovascular risk.^11^ Adverse pregnancy outcomes, such as hypertensive disorders of pregnancy and gestational diabetes, along with higher parity (number of live births), have been consistently linked to an increased risk of CVD and HF later in life.^12,13^ Recent large-scale studies have demonstrated that hypertensive disorders of pregnancy substantially increase future HF risk.^14,15^ In terms of parity, the Framingham Heart Study (*n* = 12 600) demonstrated that women with five or more live births had significantly higher risk of left ventricular dysfunction and incident HF.^16^ Similarly, data from UK Biobank indicated significantly increased HF incidence among women with 3–4 or ≥4 births compared with those with 1–2 births.^17^ However, these studies were conducted in population-based cohorts with relatively low baseline cardiovascular risk. It remains uncertain whether parity contributes independently and additively to HF risk among women with substantial pre-existing cardiovascular burden, such as those with T2DM and CAD. Accordingly, this study aimed to investigate the association between parity and the risk of HF hospitalization and mortality in women with T2DM and CAD.

## Methods

### Study design and setting

We conducted a retrospective, multicentre registry study including consecutive patients with T2DM and CAD who underwent coronary angiography (CAG) between 1 January 2005 and 31 December 2022. Data were collected from medical records by trained clinical research coordinators at 70 teaching hospitals in Japan. This study was conducted following approval by the Ethics Committee of the University of the Ryukyus, Okinawa, Japan (study ID: 104). Informed consent was obtained in the form of opt-out in each hospital.

### Patients

Eligible participants were adults (aged >20 years) with T2DM and established CAD. The diagnosis of T2DM was based on the Japanese Diabetes Care Guidelines 2016,^18^ which included the following criteria: a fasting plasma glucose level ≥126 mg/dl, a random plasma glucose level ≥200 mg/dl, or a 2-h plasma glucose level ≥200 mg/dl following a 75-g oral glucose tolerance test, with glycosylated haemoglobin A1c (HbA1c) ≥6.5%. Patients previously diagnosed with T2DM were included if their medical history and medications were consistent with the diagnosis.

CAD was defined as at least 75% stenosis in at least one major coronary artery branch, a history of acute coronary syndrome as defined by the 2003 American Heart Association^19^ and Japanese Circulation Society^20^ guidelines, or a previous percutaneous coronary intervention or coronary artery bypass grafting. Patients with malignant neoplasms and disease-free survival of <3 years were excluded.

### Data collection and variables

The enrolment date was defined as either the date of CAG, or in cases of acute coronary syndrome, the date of an outpatient visit within 3 to 6 months after CAG. Baseline data at enrolment included age, sex, height, weight, blood pressure, heart rate, medical history, treatment history, laboratory data, medication use (including antidiabetic, antihypertensive, antiplatelet, lipid-lowering agents, and diuretic agents), smoking status, and parity were collected directly from the medical records by trained clinical research coordinators.

Follow-up assessments were conducted every 6 months and included blood pressure, heart rate, laboratory results, medication updates, and cardiovascular events, which were also collected from the medical charts and referred physicians by clinical research coordinators. Follow-up continued until death or study completion. Patients who were lost to follow-up were censored on the last day with follow-up information.

### Outcome measures

The primary outcome was hospitalization for HF, defined as admission due to worsening HF requiring intravenous therapy, such as diuretics or vasodilators. The diagnosis of HF was based on the Framingham Heart Study criteria.^21^ The secondary outcomes were AMI, stroke, and all-cause mortality. AMI was defined according to the 2003 American Heart Association guidelines,^19^ and stroke was defined by the WHO MONICA Project diagnostic criteria.^22^ All events were reported by clinical research coordinators and subsequently adjudicated by an independent event adjudication committee.

### Statistical method

#### Descriptive statistics

Baseline characteristics were compared according to parity categorization. Continuous variables are expressed as mean ± standard deviation, unless otherwise specified, and categorical variables are presented as frequencies and percentages. Differences in continuous variables across parity groups were assessed using analysis of variance or Kruskal–Wallis, as appropriate. Categorical variables were compared using Pearson's χ^2^ test.

#### Survival analysis

The number of CVD events and person-years at risk were calculated by parity groups (0, 1, 2, 3, 4, and ≥5) to estimate the incidence rate per 100 person-years. The cumulative incidence of HF hospitalization, all-cause death, AMI, and stroke was estimated using the Kaplan–Meier method, with comparisons between groups (<5 vs ≥5 births) performed using the log-rank test, based on the observed threshold pattern in which an increased risk of HF was primarily evident among women with parity ≥5 (Supplementary Figure S1). Hazard ratios (HRs) and 95% confidence intervals (CIs) for cardiovascular outcomes were estimated using Cox proportional hazards models. We employed a staged adjustment approach with three pre-specified models: Model 1 adjusted for age. Model 2 adjusted for age, systolic blood pressure, LDL cholesterol (LDL-C), HbA1c, left ventricular ejection fraction (LVEF), estimated glomerular filtration rate, history of myocardial infarction, and current smoking status. Model 3 (primary fully-adjusted analysis) additionally adjusted for use of renin–angiotensin system inhibitors (angiotensin-converting enzyme inhibitors or angiotensin II receptor blockers) and sodium–glucose cotransporter 2 (SGLT2) inhibitors. The continuous variables were dichotomized by clinically meaningful reference values or median values. To evaluate the risk of first HF hospitalization, we performed a sensitivity analysis excluding women with a prior history of HF. Sensitivity analyses were performed by (i) excluding women with a prior history of HF, (ii) additionally adjusting for receipt of public assistance as a proxy for socioeconomic disadvantage, and (iii) applying a Fine–Gray subdistribution hazards model to account for the competing risk of death for HF hospitalization. The Fine–Gray model included the same covariates as the fully adjusted Cox proportional hazards model (Model 3), and results are reported as subdistribution hazard ratios (sHRs) with 95% CIs.

#### Missing data handling

Missing data were minimal across all baseline variables. Ejection fraction had the highest proportion of missing values (6.4%), followed by LDL-C (6.3%), estimated glomerular filtration rate (4.7%), and HbA1c (4.6%). All other variables had missing data below 2%. Given the low frequency of missing data, we used complete case analysis for all multivariable models rather than imputation methods.

All analyses were conducted using JMP version 18.0 (SAS Institute, Cary, NC, USA). All tests were two-sided, and a *P*-value < .05 was considered statistically significant.

## Results

**Figure 1:**
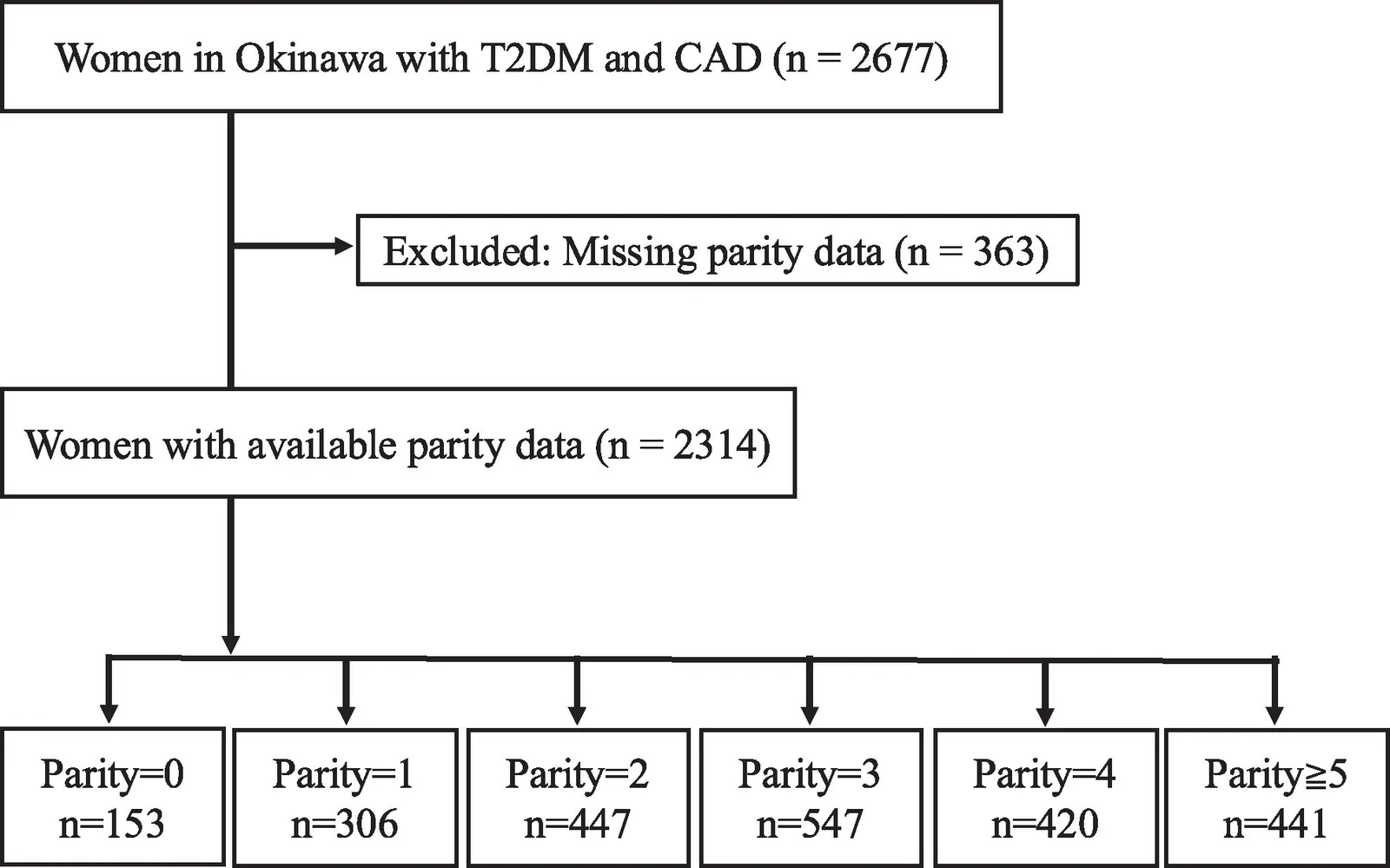
Study population. Women with confirmed parity information in Okinawa Prefecture were identified from the multicentre registry of patients with type 2 diabetes mellitus and coronary artery disease. Eligible participants were divided into six groups according to parity for analysis

A total of 11 964 patients with T2DM and coronary artery stenosis were enrolled in Japan between 1 January 2005 and 31 December 2022. Among these, 3317 (27.7%) were women, and 2677 were recruited from Okinawa Prefecture. Parity information was available for 2314 patients in Okinawa (*Figure 1*). The median follow-up period was 2297 days.

### Characteristics according to parity groups

Participants were stratified into six groups according to parity (0, 1, 2, 3, 4, and ≥5) (*Table 1*). Higher parity was associated with older age, whereas nulliparous women exhibited higher body mass index. The prevalence of smoking and receipt of public assistance were highest among nulliparous women. Although HbA1c levels were similar across groups, diabetes duration tended to be longer among women with ≥4 births. The history of HF was more frequent in both the nulliparous (22.9%) and high-parity group (≥5 births, 24.3%), although LVEF did not differ significantly among groups. No significant differences were observed in the prevalence of hypertension, cardiovascular history, systolic blood pressure, LDL-C, or aspirin use. The use of statins and SGLT2 inhibitors decreased with increasing parity, whereas calcium channel blocker and diuretic use increased with higher parity.

**Table 1:** Baseline characteristics according to parity

	All	Parity = 0	Parity = 1	Parity = 2	Parity = 3	Parity = 4	Parity ≧ 5
	(*n* = 2314)	(*n* = 153)	(*n* = 306)	(*n* = 447)	(*n* = 547)	(*n* = 420)	(*n* = 441)
**Age, y**	71.8 (±9.7)	64.1 (±12.7)	69.6 (±11.0)	70.8 (±9.5)	70.8 (±8.6)	73.6 (±8.2)	76.3 (±7.6)
**Body mass index, kg/m^2^**	26.0 (±4.6)	27.2 (±5.0)	26.3 (±4.8)	25.5 (±4.6)	25.8 (±4.7)	26.0 (±4.3)	26.0 (±4.4)
**Current smoker (%)**	181 (7.8)	27 (17.7)	36 (11.8)	36 (8.1)	43 (7.9)	17 (4.1)	22 (5.0)
**Public assistance, *n* (%)**	168 (7.3)	27 (17.7)	32 (10.5)	29 (6.5)	30 (5.5)	24 (5.7)	26 (5.9)
**LVEF (%)**	63.1 (±12.5)	61.4 (±13.5)	63.4 (±12.0)	63.0 (±12.1)	63.6 (±12.2)	63.5 (±12.0)	62.7 (±13.6)
**Duration of diabetes, y, median [iQR]**	8 [2,16]	7 [2,15]	7 [1,16]	7 [2,16]	7 [2,15]	9 [2,18]	9 [2,19]
**HbA1c-N (%)**	7.3 (±1.3)	7.3 (±1.4)	7.3 (±1.4)	7.3 (±1.3)	7.3 (±1.3)	7.3 (±1.2)	7.2 (±1.2)
**History of hypertension, *n* (%)**	1981 (85.6)	125 (81.7)	259 (84.6)	380 (85.0)	467 (85.4)	355 (84.5)	395 (89.6)
**History of myocardial infarction, *n* (%)**	512 (22.1)	42 (27.5)	59 (19.3)	114 (25.5)	118 (21.6)	87 (20.7)	92 (20.9)
**History of PCI, *n* (%)**	928 (40.1)	68 (44.4)	119 (38.9)	194 (43.4)	212 (38.8)	165 (39.3)	170 (38.6)
**History of CABG, *n* (%)**	204 (8.8)	15 (9.8)	17 (5.6)	46 (10.3)	48 (8.8)	37 (8.8)	41 (9.3)
**History of stroke, *n* (%)**	371 (16.0)	19 (12.4)	56 (18.3)	68 (15.2)	79 (14.4)	71 (16.9)	78 (17.7)
**History of cancer, *n* (%)**	149 (6.4)	14 (9.2)	13 (4.3)	35 (7.8)	31 (5.7)	29 (6.9)	27 (6.1)
**History of heart failure, *n* (%)**	442 (19.1)	35 (22.9)	53 (17.3)	83 (18.6)	98 (17.9)	66 (15.7)	107 (24.3)
**WBC count, cells/μl**	6731 (±2110)	6769 (±1741)	6889 (±2402)	6782 (±2105)	6760 (±2177)	6522 (±1971)	6719 (2061)
**Heart rate, beats/min**	78 (±13)	79 (±12)	78 (±13)	78 (±13)	78 (±13)	78 (±13)	76 (±13)
**Systolic blood pressure, mm Hg**	136 (±20)	137 (±20)	136 (±20)	134 (±19)	136 (±20)	137 (±19)	137 (±19)
**Diastolic blood pressure, mm Hg**	70 (±12)	72 (±13)	71 (±12)	69 (±12)	70 (±12)	70 (±12)	71 (±13)
**LDL-C, mg/dl**	104 (±34)	105 (±35)	106 (±36)	103 (±35)	104 (±33)	103 (±34)	103 (±33)
**eGFR, ml/min/1.73 m^2^**	57.5 (±25.6)	61.2 (±30.7)	59.8 (±27.5)	57.0 (±24.6)	60.8 (±25.7)	54.9 (±23.5)	53.5 (±24.5)
**Statin, *n* (%)**	1611 (69.6)	117 (76.5)	218 (71.2)	315 (70.5)	404 (73.9)	286 (68.1)	271 (61.5)
**Non-statin lipid-lowering agents, *n* (%)**	328 (14.2)	28 (18.3)	46 (15.0)	72 (16.1)	78 (14.3)	60 (14.3)	44 (10.0)
**Aspirin, *n* (%)**	1948 (84.2)	129 (84.3)	253 (82.7)	379 (84.8)	464 (84.8)	357 (85.0)	366 (83.0)
**Calcium blocker, *n* (%)**	1199 (51.8)	74 (48.3)	143 (46.7)	214 (47.9)	289 (52.8)	223 (53.1)	256 (58.1)
**β-blocker, *n* (%)**	784 (33.9)	59 (38.6)	95 (31.1)	166 (37.1)	181 (33.1)	123 (29.3)	160 (36.3)
**ARB, *n* (%)**	1071 (46.3)	70 (45.8)	140 (45.8)	215 (48.1)	227 (41.5)	200 (47.6)	219 (49.7)
**ACE inhibitors, *n* (%)**	456 (19.7)	33 (21.6)	52 (17.0)	86 (19.2)	121 (22.1)	83 (19.8)	81 (18.4)
**Diuretics, *n* (%)**	668 (28.9)	43 (28.1)	79 (25.8)	142 (31.8)	136 (24.9)	100 (23.8)	168 (38.1)
**Nitrates, *n* (%)**	342 (14.8)	21 (13.7)	36 (11.8)	60 (13.4)	76 (13.9)	70 (16.7)	79 (17.9)
**Nicorandil, *n* (%)**	722 (31.2)	47 (30.7)	100 (32.7)	134 (30.0)	157 (28.7)	138 (32.9)	146 (33.1)
**Insulin, *n* (%)**	532 (23.0)	38 (24.8)	63 (20.6)	98 (21.9)	129 (23.6)	99 (23.6)	105 (23.8)
**Sulfonylurea, *n* (%)**	659 (28.5)	40 (26.1)	73 (23.9)	126 (28.2)	149 (27.2)	137 (32.6)	134 (30.4)
**α-Glucosidase inhibitor, *n* (%)**	474 (20.5)	32 (20.9)	48 (15.7)	84 (18.8)	117 (21.4)	97 (23.1)	96 (21.8)
**Pioglitazone, *n* (%)**	227 (9.8)	10 (6.5)	41 (13.4)	44 (9.8)	55 (10.1)	38 (9.1)	39 (8.8)
**Biguanide, *n* (%)**	553 (23.9)	45 (29.4)	86 (28.1)	113 (25.3)	129 (23.6)	86 (20.5)	94 (21.3)
**DPP-4 inhibitor, *n* (%)**	720 (31.1)	52 (34.0)	107 (35.0)	138 (30.9)	180 (32.9)	124 (29.5)	119 (27.0)
**SGLT2, *n* (%)**	119 (5.1)	15 (9.8)	20 (6.5)	28 (6.3)	30 (5.5)	18 (4.3)	8 (1.8)

Missing data frequencies: LVEF *n* = 147 (6.4%), LDL-C *n* = 145 (6.3%), eGFR *n* = 109 (4.7%), HbA1c-N *n* = 106 (4.6%), body mass index *n* = 29 (1.3%), WBC count *n* = 30 (1.3%), heart rate *n* = 16 (.7%), diastolic blood pressure *n* = 6 (.3%), and systolic blood pressure *n* = 5 (.2%). All other variables had no missing data.

LVEF, left ventricular ejection fraction; HbA1c-N, glycated haemoglobin; PCI, percutaneous coronary intervention; CABG, coronary artery bypass grafting; WBC count, white blood cell count; LDL-C, LDL cholesterol; eGFR, estimated glomerular filtration rate; ARB, angiotensin II receptor blocker; ACE inhibitors, angiotensin-converting enzyme inhibitors.

### Incidence of HF hospitalization and all-cause death by parity

The incidence rates of HF hospitalization per 100 person-years were highest among women with parity ≥5, while mortality was elevated in both the parity ≥5 and 1 group (*Table 2*).

**Table 2:** Incidence rates of cardiovascular outcomes according to parity

Parity	HF(*n* = 477)		Death (*n* = 624)		AMI (*n* = 101)		Apo (*n* = 188)	
	events	100 person-years	events	100 person-years	events	100 person-years	events	100 person-years
**0 (*n* = 153)**	24	2.90	29	4.28	9	1.01	14	1.58
**1 (*n* = 306)**	52	3.12	86	4.74	10	.56	18	1.04
**2 (*n* = 447)**	83	3.10	92	3.10	16	.55	36	1.25
**3 (*n* = 547)**	105	3.11	121	3.22	32	.88	42	1.15
**4 (*n* = 420)**	76	2.86	129	4.36	16	.54	38	1.35
**5 (*n* = 441)**	137	4.90	157	4.85	18	.57	40	1.29

The number of events and incidence rates per 100 person-years are presented for heart failure (HF), all-cause death, acute myocardial infarction (AMI), and stroke across parity categories (0, 1, 2, 3, 4, and ≥5) among 2314 participants.

**Figure 2:**
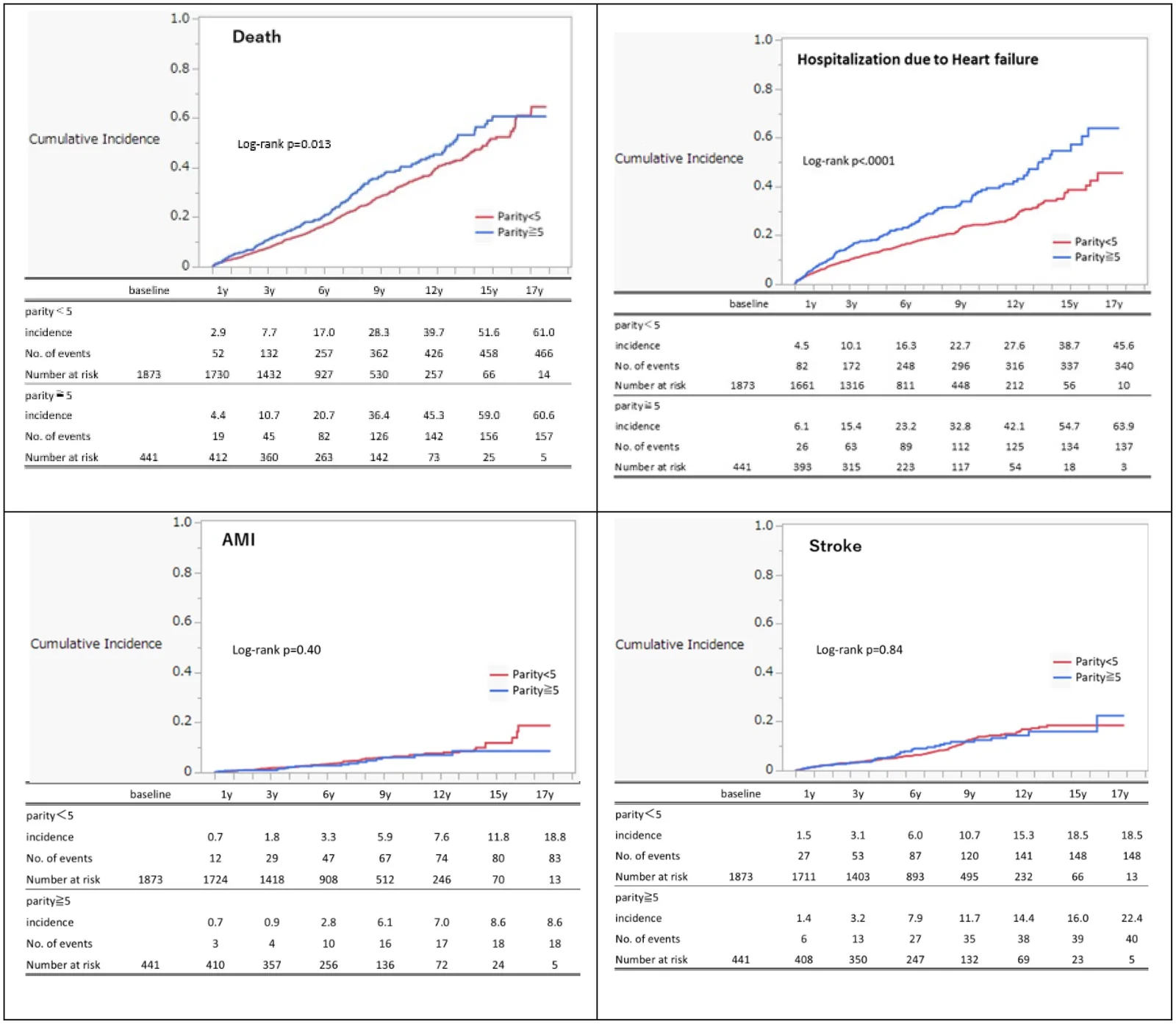
The Kaplan–Meier curves depict the cumulative incidence of death, heart failure, acute myocardial infarction, and stroke. Outcomes were compared between women with parity <5 and those with parity ≥5 using log-rank tests.

Kaplan–Meier analysis demonstrated that women with parity ≥5 births had significantly higher cumulative incidences of HF hospitalization (*P* < .0001) and all-cause death (*P* = .013) compared with those with parity <5 births (*Figure 2*). In multivariable Cox models, parity ≥5 was significantly associated with an increased risk of HF hospitalization after adjustment for age and other relevant variables (*Table 3*). Women with one birth showed significantly elevated mortality, while high parity (≥5) was not significantly associated with mortality (*Table 3*). No significant associations were found between parity and AMI or stroke (*Table 3*).

**Table 3:** Adjusted hazard ratios for cardiovascular outcome according to parity (*n* = 2314)

	Parity = 0	Parity = 1	Parity = 2	Parity = 3	Parity = 4	Parity ≧ 5
	*n* = 153	*n* = 306	*n* = 447	*n* = 547	*n* = 420	*n* = 441
**Death**						
** Model 1**	1.65 (1.13–2.40)	1.67 (1.24–2.24)	1	1.05 (.80–1.38)	1.30 (1.00–1.70)	1.37 (1.05–1.77)
** Model 2**	1.44 (.94–2.22)	1.83 (1.32–2.52)	1	1.04 (.76–1.40)	1.21 (.90–1.62)	1.21 (.91–1.60)
** Model 3**	1.43 (.93–2.19)	1.81 (1.31–2.49)	1	1.03 (.76–1.40)	1.21 (.90–1.63)	1.22 (.92–1.62)
**Heart failure**						
** Model 1**	1.00 (.63–1.58)	1.03 (.73–1.46)	1	1.02 (.76–1.36)	.89 (.65–1.22)	1.50 (1.14–1.97)
** Model 2**	1.01 (.62–1.64)	1.20 (.83–1.74)	1	1.16 (.84–1.58)	.94 (.67–1.31)	1.42 (1.05–1.91)
** Model 3**	1.02 (.63–1.65)	1.22 (.84–1.77)	1	1.17 (.85–1.60)	.94 (.67–1.32)	1.41 (1.04–1.90)
**AMI**						
** Model 1**	1.85 (.81–4.21)	1.06 (.48–2.33)	1	1.63 (.89–2.97)	1.00 (.50–2.00)	1.05 (.53–2.07)
** Model 2**	1.99 (.78–5.11)	1.12 (.46–2.75)	1	1.66 (.83–3.32)	1.04 (.47–2.29)	1.30 (.62–2.73)
** Model 3**	1.99 (.78–5.11)	1.11 (.45–2.73)	1	1.65 (.83–3.30)	1.04 (.47–2.29)	1.30 (.62–2.74)
**Stroke**						
** Model 1**	1.41 (.76–2.63)	.88 (.50–1.54)	1	.93 (.59–1.45)	1.04 (.66–1.65)	.96 (.61–1.51)
** Model 2**	1.27 (.64–2.53)	.76 (.41–1.43)	1	.82 (.51–1.33)	.95 (.59–1.54)	.79 (.49–1.28)
** Model 3**	1.26 (.63–2.51)	.76 (.41–1.42)	1	.82 (.51–1.33)	.95 (.59–1.54)	.80 (.50–1.30)

Hazard ratios (HRs) and 95% confidence intervals (CIs) are shown for death, heart failure, acute myocardial infarction (AMI), and stroke across parity categories (0, 1, 2, 3, 4, and ≥5), with parity 2 as the reference group. Model 1 was adjusted for age, Model 2 was further adjusted for systolic blood pressure (SBP), LDL cholesterol (LDL-C), glycated haemoglobin (HbA1c), left ventricular ejection fraction (LVEF), estimated glomerular filtration rate (eGFR), history of myocardial infarction, and smoking status. Model 3 was additionally adjusted for the use of renin–angiotensin system inhibitors (angiotensin-converting enzyme inhibitors or angiotensin II receptor blockers) and sodium–glucose cotransporter 2 (SGLT2) inhibitors.

The results remained consistent in sensitivity analyses that excluded women with a history of HF hospitalization (Supplementary Table S1). In an additional sensitivity analysis adjusting for receipt of public assistance as a proxy for socioeconomic status, the association between high parity (≥5 births) and HF hospitalization remained significant (HR 1.41, 95% CI 1.04–1.90). The results of this analysis are presented in Supplementary Table S2. In a competing-risk analysis using the Fine–Gray model, high parity (≥5 births) remained significantly associated with HF hospitalization (sHR 1.40, 95% CI 1.03–1.88, *P* = .029) (Supplementary Table S4).

## Discussion

### Key findings

To the best of our knowledge, this is the first study to demonstrate that a history of ≥5 births independently associated with HF hospitalization among women with T2DM and CAD. Although previous population-based studies have identified higher parity as a risk factor for HF, our study demonstrates that this association persists even in women with established CAD and T2DM, where baseline HF risk is already substantially elevated. Importantly, these associations remained significant after adjusting for traditional cardiovascular risk factors, including age, HbA1c, blood pressure, lipids, renal function, and LVEF, as well as the use of evidence-based pharmacotherapies such as SGLT2 inhibitors and renin–angiotensin system inhibitors. However, high parity was specifically associated with HF but not with all-cause mortality, AMI, or stroke.

### High parity and heart failure risk

Our findings are consistent with previous large-scale cohort studies demonstrating an association between parity and HF risk in the general population. Both the Framingham Heart Study and the UK Biobank reported higher HF risk among women with ≥4–5 births compared with those with 1–2 births.^16,17^ However, these studies focused on general populations with relatively low cardiovascular risk. This study extends these findings to a high-risk population, women with T2DM and CAD, who already have two- to five-fold greater baseline risk of HF.^2–6^ The key novel finding in this study is that even in the presence of multiple accumulated cardiovascular risk factors, parity ≥5 remains an independent predictor of HF hospitalization. These results suggest that reproductive history provides additive prognostic information beyond traditional risk factors, suggesting parity assessment should be considered in cardiovascular risk evaluation for women with T2DM and CAD.

### Mechanistic considerations

#### Treatment disparities and their limitations

In this study, SGLT2 inhibitor and statin use decreased with increasing parity (*Table 1*), likely reflecting barriers such as time constraints related to childcare, reduced healthcare access, and potential sex-based treatment gaps. However, the association between parity and HF risk persisted after adjusting for these medications and traditional risk factors (HR: 1.41, 95% CI: 1.04–1.90), suggesting that treatment disparities alone do not explain our findings. These points towards biological mechanisms related to pregnancy itself.

#### Biological mechanisms: cumulative cardiac remodelling

Although clinical left ventricular dysfunction was not evident at baseline, previous studies suggest cumulative cardiac remodelling as a mechanism. Traditionally, pregnancy-induced cardiac hypertrophy has been regarded as physiological and reversible.^23^ However, recent experimental evidence challenges this view. Experimental studies show that multiparous mice (≥5 births) exhibit persistent myocardial remodelling 15 months post-delivery, characterized by increased cardiac mass, fibrosis, up-regulated cardiac genes, and inflammatory markers.^24^ In human studies, women with HF with preserved ejection fraction and ≥3 births demonstrate impaired left ventricular diastolic reserve, increased pulmonary vascular resistance, and reduced systolic function during exercise,^25^ suggesting multi-parity-associated cardiac remodelling may pre-dispose women to HF when additional risk factors are present. Supporting this interpretation, HFpEF predominated across all parity groups among participants who developed HF, consistent with known epidemiology in diabetic women.^26^ The similar ejection fraction distribution suggests high parity affects HF risk across multiple phenotypes rather than pre-disposing to specific subtypes. This is further supported by similar ischaemic burden and blood pressure across parity groups. The HF-specific association indicates that cumulative pregnancy-induced cardiac remodelling, independent of ischaemic and hypertensive pathways, directly contributes to HF risk.

#### Contribution of pregnancy complications

Although detailed pregnancy complication data were unavailable in this study, women with higher parity likely have higher prevalence of hypertensive disorders or gestational diabetes,^27,28^ which independently increase HF risk two- to three-fold. The cumulative burden of multiple pregnancies, potentially complicated by these conditions, may amplify cardiac remodelling in women who develop T2DM and CAD. Our findings complement recent evidence from general populations showing that hypertensive disorders of pregnancy increase CVD risk.^14,15^ In our high-risk cohort with prevalent hypertension (85%–90%), the specific association with HF but not stroke or myocardial infarction despite blood pressure adjustment capturing the primary hypertension disorders of pregnancy (HDP) pathway suggests additional blood pressure-independent mechanisms. Both HDP-mediated and direct cardiac remodelling pathways likely contribute to cardiovascular risk, with relative importance varying by population risk profile.

### Low parity and mortality risk

Women with low parity showed elevated mortality, particularly those with one birth, which was consistent with previous findings.^29–31^ Nulliparous women showed similar magnitude risk without statistical significance (HR 1.43 95% CI: .93–2.19, *P* = .10), likely reflecting limited power. These mortality associations may reflect socioeconomic disadvantages^32,33^ and pre-existing comorbidities that limit reproductive capacity and also influence mortality. These factors were, however, incompletely captured by measured covariates. Notably, such associations were absent in high-parity women (HR 1.22, *P* = .17) despite the well-recognized socioeconomic burden of large families. The distinct mortality pattern i.e. mortality elevated in low parity (not high parity), HF elevated in high parity (not low parity) argues against general frailty and supports cardiac-specific mechanisms for the parity-HF association.

### Specificity of cardiovascular events: no association with acute myocardial infarction or stroke

Parity was not significantly associated with the incidence of AMI or stroke in this cohort. This lack of association may reflect several factors specific to our high-risk population. First, all participants had established T2DM and CAD, conditions that already confer substantial atherosclerotic risk. In such populations, the incremental influence of reproductive history on atherosclerotic outcomes may be attenuated by the overwhelming impact of existing risk factors. Prior studies associating parity with AMI or stroke, such as the ARIC study,^34^ were conducted in primary prevention settings where baseline cardiovascular risk was lower and reproductive history may serve as a more informative stratification factor. Second, multi-parity may primarily affect cardiac structure and function through cumulative haemodynamic stress, leading specifically to HF rather than atherosclerotic events. Traditional cardiovascular risk factors prevalent in our cohort have more direct atherosclerotic effects. This outcome-specific pattern suggests that parity is particularly important for HF risk assessment but may have limited predictive value for atherosclerotic events in populations with established T2DM and CAD.

### Strengths and limitations

#### Limitations

This study has several limitations. As a retrospective study, residual confounding from unmeasured variables cannot be excluded. Most importantly, detailed pregnancy complication data (hypertensive disorders, gestational diabetes, and peripartum cardiomyopathy) were unavailable in the retrospective registry study, as most participants were registered long after their reproductive events, with pregnancies having occurred 40–60 years before study enrolment. Advanced echocardiographic assessment between delivery and CAD diagnosis was also lacking. Comprehensive psychosocial data (chronic stress and mental health) were not available. Individual-level socioeconomic data, such as income and education, were not systematically collected; a sensitivity analysis adjusting for public assistance receipt as a proxy for low socioeconomic status yielded essentially unchanged results. Reverse causation, especially in the low-parity group, cannot be excluded. Additionally, the study’s single-region design (Okinawa) may limit generalizability to other populations. Finally, menopausal status and hormone therapy use were not systematically captured.

#### Strengths

Despite these limitations, this study has notable strengths. It represents one of the largest multicentre cohorts of women with T2DM and CAD, featuring long-term follow-up, detailed parity stratification, and independent event adjudication, providing novel insights into a population where conventional risk management is already emphasized. Moreover, comprehensive adjustment for traditional risk factors and evidence-based medications isolates parity's independent contribution. To address the high competing mortality (27% during follow-up), Fine–Gray competing-risk analyses confirmed the robustness of the primary association between high parity and HF hospitalization.

## Conclusion

High parity remains a significant and specific predictor of HF even in women with accumulated cardiovascular risk factors. Novel comprehensive HF prevention strategies beyond traditional risk factor management are needed for this population throughout their life course, considering clinical, biological, and sociological challenges.

## Clinical perspectives

From the clinical perspective, women with high parity may need additional support and regular access to care for strict risk factor management with regular echocardiographic assessment. In terms of future clinical research, prospective studies are needed with a detailed assessment of pregnancy history including pregnancy complications and cardiac function evaluation to prevent HF in high-parity women. The ‘black box’ period between childbearing and the onset of symptomatic CAD or HF warrants intensive studies.

## Supplementary Material

xvag206_Supplementary_Data

## Declarations

### Data Availability

The data that support the findings of this study are available from the corresponding author upon reasonable request and with appropriate institutional approvals.

### Pre-registered Clinical Trial Number

None supplied.
