# Supplementary material for: Parity and heart failure risk in diabetic women with coronary artery disease: a multicentre cohort study
Source: ESC Heart Fail. 2026 Aug 19;13(5):xvag206. doi: 10.1093/eschf/xvag206 (PMC13595242; doi:10.1093/eschf/xvag206)
Supplement: Supplementary file 1 [file xvag206_supplementary_data.zip › Figure S1.docx]

Figure S1. Association between parity and risk of heart failure
