# Supplementary material for: Parity and heart failure risk in diabetic women with coronary artery disease: a multicentre cohort study
Source: ESC Heart Fail. 2026 Aug 19;13(5):xvag206. doi: 10.1093/eschf/xvag206 (PMC13595242; doi:10.1093/eschf/xvag206)
Supplement: Supplementary file 1 [file xvag206_supplementary_data.zip › Supp Tables.docx]

Table S1. Adjusted hazard ratios for cardiovascular outcomes according to parity in participants without prior heart failure (n=1872)

|  | Parity=0 | Parity=1 | Parity=2 | Parity=3 | Parity=4 | parity≧5 |
| --- | --- | --- | --- | --- | --- | --- |
|  | n=118 | n=253 | n=364 | n=449 | n=354 | n=334 |
| Death |  |  |  |  |  |  |
| Model 1 | 2.03 (1.31-3.14) | 1.82 (1.29-2.58) | 1 | 1.11 (0.80-1.53) | 1.33 (0.97-1.83) | 1.19 (0.86-1.64) |
| Model 2 | 1.95 (1.19-3.17) | 1.91 (1.31-2.79) | 1 | 1.14 (0.79-1.63) | 1.21 (0.85-1.72) | 1.04 (0.73-1.49) |
| Model 3 | 1.92 (1.18-3.12) | 1.90 (1.30-2.77) | 1 | 1.13 (0.79-1.62) | 1.21 (0.85-1.72) | 1.05 (0.74-1.50) |
| Heart failure |  |  |  |  |  |  |
| Model 1 | 1.04 (0.56-1.92) | 1.32 (0.86-2.03) | 1 | 1.20 (0.83-1.73) | 0.99 (0.67-1.47) | 1.67 (1.17-2.38) |
| Model 2 | 1.00 (0.53-1.91) | 1.37 (0.87-2.16) | 1 | 1.29 (0.87-1.91) | 0.97 (0.63-1.48) | 1.53 (1.05-2.24) |
| Model 3 | 1.02 (0.53-1.95) | 1.40 (0.89-2.21) | 1 | 1.29 (0.87-1.91) | 0.96 (0.63-1.47) | 1.50 (1.02-2.19) |
| AMI |  |  |  |  |  |  |
| Model 1 | 2.06 (0.81-5.28) | 1.25 (0.53-2.97) | 1 | 1.89 (0.96-3.71) | 1.21 (0.56-2.58) | 1.28 (0.60-2.73) |
| Model 2 | 1.91 (0.70-5.24) | 1.18 (0.47-2.95) | 1 | 1.49 (0.71-3.10) | 1.00 (0.44-2.27) | 1.24 (0.57-2.70) |
| Model 3 | 1.91 (0.70-5.24) | 1.18 (0.47-2.95) | 1 | 1.49 (0.71-3.10) | 1.00 (0.44-2.27) | 1.24 (0.57-2.70) |
| Stroke |  |  |  |  |  |  |
| Model 1 | 1.57 (0.80-3.06) | 0.77 (0.41-1.45) | 1 | 0.89 (0.55-1.45) | 1.03 (0.63-1.67) | 0.81 (0.48-1.34) |
| Model 2 | 1.45 (0.70-3.00) | 0.65 (0.32-1.30) | 1 | 0.76 (0.45-1.28) | 0.93 (0.56-1.54) | 0.63 (0.37-1.08) |
| Model 3 | 1.44 (0.69-2.98) | 0.64 (0.32-1.29) | 1 | 0.76 (0.45-1.28) | 0.93 (0.56-1.54) | 0.63 (0.37-1.09) |
| Hazard ratios (HRs) and 95% confidence intervals (CIs) are presented for death, heart failure, acute myocardial infarction (AMI), and stroke across parity categories (0, 1, 2, 3, 4, ≥5), using parity 2 as the reference group among 1,872 participants without a history of heart failure. Model 1 was adjusted for age. Model 2 was further adjusted for systolic blood pressure (SBP), low-density lipoprotein cholesterol (LDL-C), glycated hemoglobin (HbA1c), left ventricular ejection fraction (LVEF), estimated glomerular filtration rate (eGFR), history of myocardial infarction and smoking status. Model 3 was additionally adjusted for the use of renin–angiotensin system inhibitors (angiotensin-converting enzyme inhibitors or angiotensin II receptor blockers) and sodium–glucose cotransporter 2 (SGLT2) inhibitors. | | | | | | |

Table S2. Hazard ratios for cardiovascular outcomes according to parity, including public assistance

|  | Parity=0 | Parity=1 | Parity=2 | Parity=3 | Parity=4 | parity≧5 |
| --- | --- | --- | --- | --- | --- | --- |
|  | n=153 | n=306 | n=447 | n=547 | n=420 | n=441 |
| **Death** |  |  |  |  |  |  |
| Model 1 | 1.58 (1.08-2.30) | 1.63 (1.21-2.19) | 1 | 1.05 (0.80-1.37) | 1.30 (0.99-1.70) | 1.37 (1.05-1.77) |
| Model 2 | 1.41 (0.92-2.18) | 1.81 (1.31-2.50) | 1 | 1.03 (0.76-1.40) | 1.20 (0.89-1.61) | 1.20 (0.91-1.60) |
| Model 3 | 1.39 (0.90-2.15) | 1.79 (1.29-2.47) | 1 | 1.02 (0.75-1.39) | 1.20 (0.90-1.62) | 1.21 (0.91-1.61) |
| **Heart failure** |  |  |  |  |  |  |
| Model 1 | 0.98 (0.62-1.55) | 1.02 (0.72-1.45) | 1 | 1.02 (0.76-1.36) | 0.89 (0.65-1.22) | 1.50 (1.14-1.97) |
| Model 2 | 1.00 (0.62-1.63) | 1.20 (0.83-1.74) | 1 | 1.15 (0.84-1.58) | 0.94 (0.67-1.31) | 1.41 (1.05-1.91) |
| Model 3 | 1.01 (0.62-1.64) | 1.22 (0.84-1.76) | 1 | 1.16 (0.85-1.60) | 0.94 (0.67-1.31) | 1.41 (1.04-1.90) |
| **AMI** |  |  |  |  |  |  |
| Model 1 | 1.73 (0.76-3.98) | 1.03 (0.46-2.27) | 1 | 1.62 (0.89-2.96) | 1.00 (0.50-2.00) | 1.05 (0.53-2.08) |
| Model 2 | 1.91 (0.74-4.94) | 1.10 (0.45-2.70) | 1 | 1.65 (0.83-3.30) | 1.04 (0.47-2.29) | 1.30 (0.62-2.74) |
| Model 3 | 1.91 (0.74-4.94) | 1.09 (0.44-2.69) | 1 | 1.64 (0.82-3.29) | 1.04 (0.47-2.28) | 1.30 (0.62-2.74) |
| **Stroke** |  |  |  |  |  |  |
| Model 1 | 1.34 (0.71-2.50) | 0.85 (0.48-1.50) | 1 | 0.92 (0.59-1.44) | 1.04 (0.66-1.64) | 0.96 (0.61-1.51) |
| Model 2 | 1.19 (0.59-2.38) | 0.74 (0.39-1.38) | 1 | 0.81 (0.50-1.32) | 0.94 (0.58-1.53) | 0.79 (0.49-1.28) |
| Model 3 | 1.18 (0.59-2.36) | 0.73 (0.39-1.37) | 1 | 0.81 (0.50-1.31) | 0.95 (0.59-1.53) | 0.80 (0.49-1.29) |
| Hazard ratios (HRs) and 95% confidence intervals (CIs) are shown for death, heart failure, acute myocardial infarction (AMI), and stroke across parity categories (0, 1, 2, 3, 4, ≥5), with parity 2 as the reference group. Model 1 was adjusted for age and receipt of public assistance., Model 2 was further adjusted for systolic blood pressure (SBP), low-density lipoprotein cholesterol (LDL-C), glycated hemoglobin (HbA1c), left ventricular ejection fraction (LVEF), estimated glomerular filtration rate (eGFR), history of myocardial infarction and smoking status. Model 3 was additionally adjusted for the use of renin–angiotensin system inhibitors (angiotensin-converting enzyme inhibitors or angiotensin II receptor blockers) and sodium–glucose cotransporter 2 (SGLT2) inhibitors. | | | | | | |

Table S3. Distribution of heart failure phenotypes according to parity

|  | parity=0  n=153 | parity=1  n=306 | parity=2  n=447 | parity=3  n=547 | parity=4  n=420 | parity≧5  n=441 |
| --- | --- | --- | --- | --- | --- | --- |
| Heart Failure Hospitalization (n) | 24 | 49 | 77 | 100 | 71 | 133 |
| HFrEF, n (%) | 5 (20.8) | 6 (12.2) | 5 (6.5) | 13 (13.0) | 7 (9.9) | 15 (11.3) |
| HFmrEF, n (%) | 5 (20.8) | 2 (4.1) | 13 (16.9) | 15 (15.0) | 11 (15.5) | 14 (10.5) |
| HFpEF, n (%) | 14 (58.3) | 41 (83.7) | 59 (76.6) | 72 (72.0) | 53 (74.7) | 104 (78.2) |
| HFrEF, heart failure with reduced ejection fraction (EF <40%); HFmrEF, heart failure with mildly reduced ejection fraction(EF 40–49%); HFpEF, heart failure with preserved ejection fraction (EF ≥50%); EF, ejection fraction. | | | | | | |

Table S4. Sub distribution Hazard Ratios (SHRs) for heart failure according to parity

|  | Number of live births | | | | | |
| --- | --- | --- | --- | --- | --- | --- |
|  | 0 | 1 | 2 | 3 | 4 | ≧5 |
|  | n=153 | n=306 | n=447 | n=547 | n=420 | n=441 |
| Heart failure |  |  |  |  |  |  |
| Crude | 0.86  (0.55-1.34) | 0.95  (0.67-1.35) | 1 | 1.02  (0.76-1.36) | 0.89  (0.65-1.21) | 1.54  (1.17-2.02) |
| Model 1 | 0.92  (0.58-1.43) | 0.96  (0.68-1.36) | 1 | 1.02  (0.76-1.36) | 0.86  (0.63-1.18) | 1.46  (1.11-1.93) |
| Model 2 | 0.99  (0.61-1.61) | 1.10  (0.76-1.61) | 1 | 1.17  (0.86-1.61) | 0.93  (0.66-1.31) | 1.42  (1.05-1.91) |
| Model 3 | 0.99  (0.61-1.62) | 1.11  (0.76-1.62) | 1 | 1.18  (0.86-1.62) | 0.93  (0.66-1.30) | 1.40  (1.03-1.88) |
|  |  |  |  |  |  |  |
| Sub distribution Hazard Ratios (SHRs) and 95% confidence intervals (CIs) are shown for heart failure across parity categories (0, 1, 2, 3, 4, ≥5), with parity 2 as the reference group. Model 1 was adjusted for age, Model 2 was further adjusted for systolic blood pressure (SBP), low-density lipoprotein cholesterol (LDL-C), glycated hemoglobin (HbA1c), left ventricular ejection fraction (LVEF), estimated glomerular filtration rate (eGFR), history of myocardial infarction, and smoking status. Model 3 was additionally adjusted for the use of renin–angiotensin system inhibitors (angiotensin-converting enzyme inhibitors or angiotensin II receptor blockers) and sodium–glucose cotransporter 2 (SGLT2) inhibitors. | | | | | | |
